# Effects of Using Effective Microorganisms (EMs) and Zeolite on the Development of Productivity and Air and Litter Quality in Broiler Chicken Rearing

**DOI:** 10.3390/ani16060911

**Published:** 2026-03-13

**Authors:** Ewa Sosnówka-Czajka, Iwona Skomorucha, Dorota Wojtysiak, Magdalena E. Skalska, Patrycja Adamczyk

**Affiliations:** 1 Department of Poultry Breeding, National Research Institute of Animal Production, 32-083 Balice, Poland; iwona.skomorucha@iz.edu.pl (I.S.); patrycja.adamczyk@iz.edu.pl (P.A.); 2 Department of Genetics, Animal Breeding and Ethology, University of Agriculture in Krakow, Al. Mickiewicza 24/28, 30-059 Krakow, Poland; 3 Department of Medical Physics, M. Smoluchowski Institute of Physics, Faculty of Physics, Astronomy and Applied Computer Science, Jagiellonian University, 30-348 Krakow, Poland; magda.skalska@uj.edu.pl; 4 Centre for Theranostics, Jagiellonian University, 31-501 Krakow, Poland

**Keywords:** broiler chicken, zeolite, effective microorganisms (EMs), litter quality, ammonia, productivity, meat quality, footpad dermatitis

## Abstract

The growing greenhouse gas emissions from agricultural production are increasing the pressure on poultry producers to use practices that reduce the environmental impact of farms, mainly by reducing ammonia emissions. The study evaluated the effect of adding effective microorganisms (EMs) and zeolite to litter on productivity, meat quality, selected physiological parameters, and air and litter quality in broiler chickens. In the experimental group, zeolite was applied at 3 kg/10 kg to the litter, and it was sprayed with a solution of EMs and water in a 1:4 ratio. Spraying was repeated weekly until the end of the production cycle. The litter additives reduced the moisture content and pH of the litter (*p* < 0.05) in the experimental group. This group also showed lower air humidity (*p* < 0.01) and reduced levels of ammonia and carbon dioxide compared to the control group (*p* < 0.05). A positive effect of litter additives on production results and the health of broiler chickens was noted. In summary, the addition of 3 kg/10 kg of zeolite to the litter, combined with spraying the litter with a 1:4 solution of EMs and water, can contribute to reducing the emission of harmful gas admixtures generated on broiler farms without negatively affecting production efficiency.

## Simple Summary

Poultry farming has negative environmental impacts, such as greenhouse gas emissions into the atmosphere and excessive production of ammonia (NH_3_), which also adversely affects the health and production parameters of birds. Various preparations are used as litter additives to neutralize ammonia and other gas emissions and improve the performance and welfare of broiler chickens. Our own research examined the synergistic effect of zeolite and probiotics (effective microorganisms—EMs) as litter additives on productivity, meat quality, selected physiological parameters, and air and litter quality in broiler chicken rearing. The results showed that the litter additives used can improve the environmental conditions in the broiler house (litter moisture, pH, and air parameters), as well as production results and bird health. The developed technology for broiler chicken rearing using zeolite and probiotics as litter additives can therefore contribute to reducing the emissions of harmful gas admixtures generated on broiler chicken farms without negatively affecting production efficiency.

## 1. Introduction

According to the adopted provisions, by 2030, the European Union must, among other things, reduce greenhouse gas (GHG) emissions from agriculture and increase carbon dioxide (CO_2_) sequestration, while improving biodiversity and ensuring high food quality [1]. GHG emissions into the atmosphere from agriculture, forestry, and fisheries in 2023 accounted for 13.5% of total GHG emissions in the European Union [2]. Agricultural activity affects greenhouse gas emissions by changing carbon (C) and nitrogen (N) processes in the environment. On the other hand, GHG emissions from agriculture can be reduced, among other things, through the use of climate-friendly agricultural practices aimed at reducing harmful gas emissions into the environment [3].

The growing demand for animal protein products has led to the intensification of the agricultural industry and the emergence of, among other things, industrial farms, including poultry farms. In turn, the high concentration of poultry production in a relatively small area leads to the generation of huge amounts of post-production waste, chicken droppings and manure, and is also associated with significant emissions of ammonia (NH_3_) into the atmosphere [4]. Broiler chicken droppings contain large amounts of uric acid (approx. 80%), which is hydrolyzed into urea through aerobic decomposition and then converted to ammonium (NH_4_^+^) by the urease enzyme found in the droppings [4]. NH_3_ then volatilizes as a gas and is released into the atmosphere from the litter, while nitrous oxide (N_2_O) is released through nitrification and denitrification processes [5]. In addition, broiler chickens emit CO_2_ during respiration, hydrolysis of uric acid/urea from excreta, and aerobic/anaerobic decomposition of litter, while methane (CH_4_) is released through anaerobic decomposition [6]. In European countries, average annual emission rates from broiler chicken farms vary considerably: from 0.06 to 0.45 g/day/broiler chicken for NH_3_, from 0 to 46 mg/day/broiler for N_2_O, from 55.2 to 98.4 g/day/broiler for CO_2_, and from 0 to 50 mg/day/broiler for CH_4_ [5,7,8].

Litter with droppings is a significant source of NH_3_ emissions, as it contains high levels of nitrogen, accounting for 3.8% of dry matter [9]. NH_3_ emissions pose a serious risk to human health and the environment, with approximately 90% of global NH_3_ emissions coming from agriculture-related activities [10]. Emissions of ammonia not only represent a loss of the valuable nutrient nitrogen (N), but also serve as a precursor to fine particulate matter (i.e., with an aerodynamic diameter ≤ 2.5 μm; PM_2.5_) that affects respiratory health [11], and indirectly serve as a precursor to the potent greenhouse gas N_2_O [5,12].

Environmental problems in the commercial poultry industry are most often associated with high humidity in the poultry house and the quality of litter and excreta, which affect the health, welfare, and performance of birds [13]. The growth of uricolytic microorganisms in litter, combined with high humidity of 40–60%, can cause increased emissions of harmful gas admixtures, particularly ammonia, and litter loses its beneficial gas absorption properties. Ongoing decomposition processes combined with bird droppings cause increased ammonia production [14].

There are studies indicating that NH_3_ release from poultry houses can be minimized by dietary manipulations without affecting the birds’ production performance [15]. According to Brink et al. [16], the N content in broiler excreta and litter is influenced by the composition of the feed and the efficiency with which the bird converts N in the diet into animal protein. A reduction in the amount of ammonia in litter can be achieved by increasing the digestibility of nutrients and inhibiting the activity of the enzyme uricase in excreta [17].

On the other hand, numerous studies also concern the use of additives affecting ammonia emissions in litter: disinfectants [14], aluminum chloride [18], calcium peroxide [19], aluminum sulfate [20,21,22], urease inhibitor [22], biochar [22], probiotics [23,24], humic substances [24], and nanosilver [25].

Nitrogen excretion by poultry is relatively high, ranging from 1.01 to 4.80 g/day, depending on the age of the bird. Therefore, under production conditions, the concentration of NH_3_ in the air often exceeds the recommended standards. One method of reducing ammonia emissions from animal production is to add chemical preparations to the litter that neutralize ammonia and other gas emissions and also have bactericidal and deodorizing effects [18]. An increasingly used alternative to chemical compounds are natural preparations, e.g., aluminosilicates (bentonite, zeolite, or dolomite) [26]. Aluminosilicates are naturally occurring zeolites that have a three-dimensional structure composed of aluminum oxide and silica. Due to their physicochemical properties, such as ion exchange, absorption, and catalytic abilities, they are used in poultry production to reduce ammonia emissions and the intensity of odors emitted [13]. Natural zeolites, due to their global availability in various structures, continuous exploitation, properties such as ease of ion exchange and adsorption, as well as environmental friendliness and low cost, are used, among other things, in water and wastewater treatment, and water and gas absorption [27]. These minerals also contain sodium and calcium, and in some cases elements such as barium, magnesium, manganese, and many others. Due to their absorption properties, zeolites are used, among other things, as an additive to animal bedding materials. Their presence makes the substrate better at absorbing moisture and odors, and slows down the rate of bacterial growth. Natural zeolites have no harmful effects on humans, animals, or the environment [28]. Acidogenic materials reduce the pH of excreta, causing NH_3_ to protonate to NH_4_^+^, which is less volatile, while zeolite also shows a strong preference for binding nitrogen cations such as NH_4_^+^, resulting in lower NH_3_ emissions [21,29].

Another alternative is probiotics, which, when added to feed or litter, can have a beneficial effect on the health, production performance, and welfare of broiler chickens, as well as on environmental conditions [17,30].

Litter quality is an important factor shaping the environment in poultry houses. Currently, many methods are used to optimize the physicochemical parameters of poultry litter [19]. However, the use of litter additives to reduce moisture and ammonia emissions is not always effective. Consequently, there is still a need to develop new methods for reducing NH_3_ and greenhouse gas emissions in poultry facilities.

Despite the extensive literature on the use of various litter additives, including zeolite, to improve the microclimate in broiler houses, there is little information on the simultaneous use of several preparations and their synergistic effects. Due to its effectiveness, zeolite is used relatively often as a litter additive and is readily available on the market. On the other hand, probiotics, mainly effective microorganisms (EMs), are perceived by farmers and consumers as safe for animals and beneficial to their productivity, which is why they are widely used in poultry farming. Taking these factors into account, the research hypothesis assumed that the synergistic effect of adding zeolite and effective microorganisms to litter would have a positive effect on the pH and moisture content of the litter, which would translate into a reduction in ammonia emissions. Another research assumption was that better environmental conditions and colonization of the birds’ living environment by probiotic bacteria would improve the production performance and health of broiler chickens and could also affect meat quality. Therefore, based on this experience, it was decided to investigate the synergistic effect of adding zeolite and effective microorganisms to the litter. In order to verify the above hypothesis, studies were conducted to assess the effect of adding effective microorganisms (EMs) and zeolite on litter on productivity, meat quality, selected physiological parameters, and air and litter quality in broiler chicken rearing.

## 2. Materials and Methods

The study was approved by the 1st Local Ethical Committee for Animal Experiments in Kraków (no. 924/2024, 10 October 2024).

### 2.1. Animals and Experimental Design

The experimental material consisted of 480 one-day-old Ross 308 slaughter cockerels from the Poultry Hatchery in Wolsztyn (Poland). On the first day of life, the chicks, after being weighed and marked with chick tags, were allocated to two groups: control (CON) and experimental (zeolite + EM). In each group, 240 birds were randomly divided into six subgroups—pens (40 birds per subgroup—pen). Slaughter chickens from the control and experimental groups were kept in two separate rooms with the same volume of 82.8 m^3^ with electronic microclimate control using a PL-9000 poultry computer from STIENEN BE (Nederweert, The Netherlands). The dimensions of the pens (replicates), in which the broilers were kept were as follows: 2.7 m × 1.2 m = 3.24 m^2^. The birds were reared on sawdust litter at a stocking density of 33 kg/m^2^ [31] for a period of 35 days. In each pen (replicate), in both the control and experimental groups, 10 kg of litter was scattered, creating a layer approximately 10 cm thick. In the experimental group, before placing the chicks, a single addition of zeolite (0.5–1 mm) in the amount of 3 kg/pen (replicate) was applied to the litter, which was evenly spread over the litter material and then raked in order to mix it with the litter. The litter in the experimental group was also sprayed with a solution of effective microorganisms (EMs) and water in a ratio of 1:4. Spraying was repeated weekly until the end of the production cycle.

The zeolite used in the experiment was purchased from Grafmind (Bielsko-Biała, Poland) and consisted mainly of clinoptilolite (>80%). The chemical composition of the mineral was as follows: silicon dioxide (SiO_2_) 68.5%, potassium oxide (K_2_O) 2.8%, sodium oxide (Na_2_O) 0.75%, aluminum oxide (Al_2_O_3_) 12.5%, iron oxide (Fe_2_O_3_) 1.3%, titanium dioxide (TiO_2_) 0.2%, calcium oxide (CaO) 4%, and magnesium oxide (MgO) 0.9%. The ion exchange capacity was 1.2–1.5 mol/kg.

The EM probiotic was purchased from Greenland Technologia EM (Janowiec n/Wisłą, Poland). It was produced on the basis of fermented sugar cane molasses. Fermentation was carried out with the participation of live and active probiotic microorganisms such as *L. casei*, *L. parafarraginis*, *L. farraginis*, *L. buchneri*, *L. plantarum*, *Bacillus subtilis*, and *Saccharomyces cerevisiae* (Effective Microorganisms (EM^®^) Technology). The total content of active lactic acid bacteria was at least 1.5 × 10^6^ CFU/mL.

The temperature inside the broiler house was 33 °C in the first few days and was then lowered gradually to 20–18 °C in the 5th week of rearing, while humidity was maintained at 65%. The light program used was: 23 h of light/day to the 7th day, and from the 8th day, 20 h of light/day. Light intensity during the laying period was maintained at 20 lux. The same light program was used in all groups.

The birds were fed a standard 4-phase feed for broiler chickens: starter, grower 1, grower 2, and finisher (Table 1). Feed and water were manually delivered to feed hoppers and bell drinkers, respectively. Throughout the rearing period, the broiler chickens had free access to feed and water drinkers.

### 2.2. Data, Sample Collection and Laboratory Analyses

During the rearing of broiler chickens, air quality (temperature, humidity, NH_3_, CO_2_, CH_4_, N_2_O, and CO) was tested twice a week in the control and experimental broiler houses. Air parameters were measured using a GASMET GT5000 Terra device (Vantaa, Finland). Measurements were taken in each pen (subgroup) at 5 points diagonally at the height of the birds.

Every week, litter was sampled from each pen from 5 different locations: 2 locations in the proximity of the feeders and drinkers, 2 locations away from the feeders and drinkers and 1 location in the center of the pen. From each of the 5 sampling locations, approximately 50 g of litter was collected from the surface to the base of the litter layer to determine the concentrations of moisture and pH.

Litter moisture was determined gravimetrically. Litter samples were separated and weighed before and after drying in a 06-WGLL-125BE drier (CHEMLAND, Stargard, Poland) with forced air circulation at 105 °C for 24 h [32]. A Radwag PS600.X2 scale (Radwag Balances and Scales, Radom, Poland) was used to weigh the samples. Moisture content was determined using the wet and dry weight of the sample [32]. Based on the obtained moisture content values of the litter from each subgroup, the average moisture content of the litter for each subgroup was calculated, and then the average for each study group for the entire duration of the experiment.

The pH of the litter was measured using a Dramiński PHG acidity tester (Dramiński, Sząbruk, Poland) and an EC-FG 73905 probe. Five grams of litter material was weighed from the collected samples; then, the weighed litter was poured into 25 mL of deionized water and thoroughly mixed. After waiting 30 min, the pH of the litter and water mixture was measured according to the manufacturer’s instructions.

During the experiment, the weight of the birds was individually monitored, as was the feed consumption and feed conversion ratio (in subgroups) per kg of weight gain, and mortality. The body weight of the chickens was monitored once a week. Birds were weighed individually on a RADWAG WPT/F-30C scale (Radwag Balances and Scales, Radom, Poland). Feed conversion efficiency (kg per kg weight gain) was calculated by measuring the feed consumption and body weight. The mortality of the birds was presented as a total of death losses for each group.

On day 35 of rearing, 20 birds, whose body weights were similar to the average from each group, were slaughtered by decapitation, after prior stunning with a KOMA STZ 6 (KOMA, Świdnica, Poland) electric stunner. Fifteen minutes post-mortem, the pH of breast and leg muscles (pH_15_) was measured using a CyberScan 10 pH meter (Cole-Parmer, Vernon Hills, IL, USA) and an EC-FG 73905 electrode, which was followed by standard post-slaughter processing (scalding, defeathering, and evisceration). The carcasses (with neck and giblets) were placed in a cold store at 4 °C. After 24 h, they were again analyzed for the acidity of breast and leg muscles (pH_24_). The chilled carcasses were subjected to simple carcass analysis according to the method described by Ziołecki and Doruchowski [33].

Meat quality analysis (pH, drip loss, water-holding capacity, and color) was determined in 20 samples of breast muscles and 20 of leg muscles per group. The color of the dissected breast and leg muscles was determined with a Minolta CR 310 (Konica Minolta, Tokyo, Japan) reflection chroma meter set to the CIE L*a*b* system, where L* represents lightness, a* redness, and b* yellowness. Water-holding capacity (WHC) was determined by the Grau and Hamm method [34]. The measurement of meat juice loss was performed after 24 and 48 h of cooling at 4 °C, as well as after 30 days of freezing the samples at −20 °C. For this purpose, samples weighing approx. 80 g (e = 0.001 g) were taken from the left breast muscle (*M. pectoralis maior*) and left thigh, placed in sealed containers, and stored in a cold room for 24 h. The samples were then weighed again, and the juice loss was determined as a percentage of the initial weight loss [35]. The procedure was repeated after 48 h of cooling and 30 days of freezing the meat samples.

In the last week of rearing, a poultry welfare specialist assessed the condition of the legs of slaughter chickens for footpad dermatitis (FPD) and changes in the hock burns. For this purpose, 60 birds were randomly selected from the group (10 birds from each subgroup) and pathological changes on the footpads and hocks were assessed using a scoring range from 0 to 2, in accordance with Dowsland’s method [36].

The results were presented as averages of the scores for the tested group and as a percentage of birds with scores of 0, 1, and 2.

In the 5th week of rearing, blood was collected by decapitation from 10 birds per group, after prior stunning with a KOMA STZ 6 (KOMA, Świdnica, Poland) electric stunner. Birds were randomly selected from the groups. Blood was collected into tubes with EDTA (ethylenediaminetetraacetic acid) salt anticoagulant and lithium heparin. The ammonia level in the blood collected on EDTA was determined on an MNCHIP chemical analyzer (MNCHIP, Tianjin, China) using an ammonia test profile reagent rotor (Tianjin, China).

Blood collected into lithium heparin tubes was centrifuged (MPW-52, MPW Med. Instruments, Warsaw, Poland) and plasma separated by centrifugation was pipetted into Eppendorf tubes. Plasma levels were determined for alanine aminotransferase—ALAT (A6424-125), aspartate aminotransferase—ASPAT (A6461-125), alkaline phosphatase—ALP (F6406-075), lactate dehydrogenase—LDH (MI41214), bilirubin (MI1001047), glucose (G6620-100), calcium—Ca (MI1001065), phosphorus—P (MI1001155), iron—Fe (MI1001247), creatinine (MI1001111), urea (M6652-125), uric acid (MI41001), cholesterol (MI41021), high-density lipoprotein HDL (H6421-160), low-density lipoprotein LDL (MI41214), triglycerides (T6630-100), sodium—Na (DZ 114B-K01) and potassium—K (DZ 113C-K01). The biochemical parameters levels were analyzed with a Mindray BS-120 biochemistry analyzer using Alpha Diagnostics (Warsaw, Poland), Spinreact (Girona, Spain) and Diazyme (Poway, CA, USA) reagent kits.

### 2.3. Statistical Analysis

Prior to testing, the Shapiro–Wilk test was used to assess the normality of distribution. The test confirmed that the data analyzed conformed to a normal distribution. The experimental units were meat (breast and leg muscle) samples (pH, drip loss, WHC, and color), birds (body weight, dissection analysis, condition of feet, and blood), and replicate (subgroup) to obtain data on litter and air quality, and the feed conversion ratio. The statistical calculations were made with Statistica 13.3 PL software (StatSoft Inc., Tulsa, OK, USA), and the results obtained were presented as mean values ± SD (Standard Deviation). The data were analyzed using the statistical method of the *t*-test for independent samples. Effects were considered significant at a probability of *p* < 0.05 and *p* < 0.01.

## 3. Results

### 3.1. Litter Quality and Air Parameters

Table 2 presents the results of pH and litter moisture during broiler chicken rearing. In the third, fourth, and fifth weeks of the experiment, lower litter pH was found in the zeolite + EM group at *p* < 0.01, *p* < 0.01, and *p* < 0.05, respectively. In the same weeks, lower litter moisture content was also recorded in the experimental group compared to the control group at *p* < 0.05 in weeks 3 and 4 and at *p* < 0.01 in week 5 of the experiment. During the rearing of broiler chickens, the CON group showed an average pH value higher by 0.3 and average litter moisture higher by 11.27% compared to the zeolite + EM experimental group at *p* < 0.05.

Table 3 shows the average air quality inside the broiler houses for the entire rearing period. Analysis of air parameters revealed lower humidity and lower air concentrations of NH_3_ and CO_2_ in the room where the zeolite + EM group was kept, at *p* < 0.01, *p* < 0.05, and *p* < 0.05, respectively.

### 3.2. Productivity and Meat Quality of Broiler Chickens

On days 9, 16, and 35 of the experiment, broiler chickens from the zeolite + EM group had a higher body weight at *p* < 0.05, *p* < 0.01, and *p* < 0.05, respectively (Table 4). Better feed conversion (*p* < 0.05) per 1 kg of body weight gain and a lower percentage of dead chickens (*p* < 0.05) were also observed in the experimental chickens (Table 4).

Table 5 presents the results of the slaughter analysis of the birds. A significant difference (*p* < 0.05) was found in the percentage of breast muscle in the carcasses. The other parameters of the slaughter analysis were similar in both groups.

The pH of the breast muscles, measured 15 min after slaughter, showed a lower degree of acidity in the control group of slaughter chickens at *p* < 0.01 (Table 6).

No differences were found between the groups in the measurement of meat juice loss performed after 24 and 48 h of cooling at 4 °C, as well as after 30 days of freezing the samples at −20 °C (Table 7). In the case of broilers from the experimental group (zeolite + EM), a higher WHC index of breast muscles was recorded at *p* < 0.01 (Table 7).

Table 8 shows the color of the breast and leg muscles of broiler chickens. A difference between the groups was observed in the case of breast muscles in the b* value at *p* < 0.05.

### 3.3. Condition of Feet and Biochemical Blood Parameters

Table 9 shows the health status of the feet of broiler chickens. Birds from the zeolite + EM group had better foot condition. This group had a lower average score for FPD (footpad dermatitis) and changes in the hock burns compared to the CON group (*p* < 0.05). The zeolite + EM group also had a higher percentage of birds showing no changes to hock burns and no birds with severe hock burns (*p* < 0.01).

The levels of the blood parameters tested in slaughter chickens are presented in Table 10. Lower concentrations of ammonia (*p* < 0.01), ALAT (*p* < 0.05), bilirubin (*p* < 0.05), Ca (*p* < 0.01), creatinine (*p* < 0.01), total cholesterol (*p* < 0.05), and HDL (*p* < 0.05), as well as higher levels of triglycerides (*p* < 0.01), were noted in the blood of experimental birds compared to birds from the control group.

## 4. Discussion

Gas emissions from poultry litter cause production problems for farmers as well as environmental problems, contributing to climate change and air quality deterioration [37]. Zeolites are crystalline minerals with unique properties such as hygroscopicity, cation exchange capacity, and ion adsorption. Due to their ammonium adsorption and water absorption properties, the use of zeolites in animal husbandry can reduce NH_3_ levels and humidity in livestock buildings [38]. Similar relationships were found in our own research. The addition of zeolite + EM to bedding was shown to have a beneficial effect on its quality: a reduction in moisture content by approximately 11 percentage points and pH by 3.3% compared to the control group. Similar results were obtained by Ezenwosu et al. [39] when applying zeolite in various concentrations to wood sawdust bedding, observing an improvement in bedding quality in terms of moisture content and pH, among other aspects. Elsherbeni et al. [13] also obtained similar results when applying zeolite in concentrations of 0.5, 1, and 1.5 kg/m^2^ to sawdust bedding, observing a highly significant effect of zeolite on reducing the pH and moisture content of the litter. According to Eleroglu and Yalcin [28], zeolite has a beneficial effect on litter moisture content. Park et al. [40] demonstrated a reduction in litter moisture content as a result of adding 2% zeolite to the litter. However, Karamanlis et al. [41], when applying a natural zeolite—clinoptilolite—in an amount of 2 kg of zeolite/m^2^ to sawdust litter, did not find any effect on litter moisture. Loch et al. [42] also did not demonstrate any effect of a 5% zeolite additive to litter on its moisture.

The pH of the litter used in poultry production ranges from 8 to 10, while the pH of dry litter is approximately 7 [43,44]. Ammonia emissions decrease when the pH of the litter is below 7, but increase significantly above 8 [45]. Rappert and Müller [46] suggested that increased litter pH accelerates the microbial decomposition of nitrogen compounds into ammonia. The action of an enzyme called uricase, which catalyzes the degradation of uric acid to ammonia, peaks at pH 9, thereby increasing ammonia production from litter [47]. Therefore, maintaining the pH of litter below neutral is particularly important, as alkaline conditions promote uricase activity and ammonia volatilization [48]. Under lower pH conditions, the equilibrium between ammonium (NH_4_^+^) and ammonia (NH_3_) shifts toward the non-volatile form NH_4_^+^, reducing the release of ammonia into the environment in the livestock building [49]. According to Nahm [47], the order of the most important factors influencing ammonia formation is litter pH > temperature > moisture content, with total ammonia binding occurring below pH 4. Low litter pH is not associated with negative effects on chickens; on the contrary, it can have a beneficial effect on productivity. According to Line [50], lower litter pH results in a reduction in the level of pathogens in the litter and of dead birds. Ezenwosu et al. [39], using zeolite in litter at a rate of 400 g and 600 g/kg, obtained a decrease in litter pH to 4.34 and 3.47, respectively, while at the same time achieving the highest final body weight of broiler chickens with the best feed conversion in these groups. In turn, Rothrock et al. [51] used an additive to the litter that lowered its pH, which in turn reduced infections and ultimately increased the birds’ production capacity.

Reszka et al. [52] found that EMs in contact with organic matter cause an increase in the production of short-chain fatty acids, which lower the pH, exerting an antibacterial effect by selectively blocking the colonization of pathogens (adhesion). In turn, Park and Sun [53] found a beneficial effect of adding probiotics to feed on reducing litter moisture. Our own research also found lower litter moisture and pH in the group where zeolite + EM was added.

Broiler chicken production is a significant source of gas emissions [22]. An important factor determining NH_3_ emissions from animal waste is pH [15]. Lower pH limits the conversion of ammonium nitrogen to NH_3_, which reduces ammonia emissions into the atmosphere [54]. In our own studies, lower litter pH also reduced the level of ammonia in the air by approximately 54% compared to the control group. Ezenwosu et al. [39] also demonstrated a relationship between lower litter pH and lower ammonia production from litter. On the other hand, Brink et al. [16] showed that low litter moisture leads to reduced ammonia emissions. In our own research, the addition of zeolite + EM to the litter reduced its moisture content, which also contributed to the reduction in ammonia. The effect of zeolite added to litter on reducing ammonia production was also demonstrated by Li et al. [48], whereby a single addition of 2.5%, 5%, and 10% zeolite resulted in a total cumulative reduction in NH_3_ emissions of 20%, 50%, and 77%, respectively. Schneider et al. [39] found that adding 10% natural zeolite (clinoptilolite) to the total weight of sawdust litter reduced the moisture content and pH of the litter and ammonia volatilization by up to 32%. In contrast, the use of natural zeolite (clinoptilolite) at a rate of 2344 g/m^2^ resulted in a 28% reduction in ammonia emissions [55]. Yayli and Kilic [56] demonstrated a 40–45% reduction in NH_3_ levels by adding 5% zeolite to the litter. Sheng et al. [57] found a 68% decrease in ammonia emissions when 20% zeolite was added to litter for broiler chickens. Emam et al. [58] used a 20% zeolite additive to litter and demonstrated its beneficial effect on reducing ammonia levels and air humidity. The use of zeolite in concentrations of 8% and 11% in litter for broiler chickens reduced NH_3_ emissions by 20% and 33%, respectively [59]. According to Nuernberg et al. [60], zeolites effectively adsorb ammonia released from litter, with its effectiveness depending on the origin of the mineral. However, the use of the natural zeolite clinoptilolite in the amount of 2 kg of zeolite/m^2^ in sawdust litter had no effect on ammonia emissions into the air of broiler houses [41].

Eglite et al. [61] added 5 g/m^2^ of a mixture of lactic acid bacteria comprising *Lactobacillus farcimins* CNCM-I-3699 (7.8 × 10^6^ GU/g) and *Lactobacillus rhamnosus* CNCM-I-3698 (7.8 × 10^6^ GU/g) to the litter every week and found that the probiotics used effectively reduced ammonia levels in the poultry house. In contrast, Ahmed et al. [62] found that *Bacillus amyloliquefaciens* KB3 reduced NH_3_ emissions. On the other hand, Park and Sun [53] found no changes in ammonia levels under the influence of *B. velezensis* CE 100. Gałęcki et al. [63] showed that a deodorizing biopreparation consisting of a mixture of six bacterial strains and a mineral sorbent (perlite + bentonite) applied to the litter for laying hens once a week had a positive effect on reducing ammonia concentration and air humidity in the poultry house. Similarly, in our own research, the addition of zeolite + EM to the litter reduced the level of NH_3_ and air humidity in the broiler house. Joshi et al. [64] found that EMs reduce unpleasant odors on farms caused by trimethylamines and ammonia, i.e., they reduce ammonia emissions from feces. Effective microorganisms used in poultry houses, added directly to droppings or litter, reduce odor emissions, mainly ammonia [65,66]. Wan et al. [67] studied the effects of EM inoculation on chicken manure and showed, among other things, a significant effect of EMs on reducing NH_4_^+^ content, which directly translates into reduced ammonia emissions. According to Li and Ni [68], the use of EMs on poultry farms has enormous potential for reducing unpleasant odors. The authors studied the effect of adding EMs to drinking water and feed for poultry and found that EMs significantly reduced the unpleasant odor of poultry manure, which was mainly associated with a significant decrease in NH_3_ levels (from 42 to 70% compared to the control group). Addeo et al. [69], evaluating in vitro the effect of different doses of EMs, alone or with zeolite, on poultry manure, demonstrated that the use of EMs with the addition of zeolite is an effective tool for reducing NH_3_ concentration.

Although NH_3_ is the largest atmospheric pollutant in relation to poultry production, poultry farms are also a source of emissions of the three most important greenhouse gases, CH_4_, CO_2_, and N_2_O, which contribute to global climate change. In our own research, better litter quality directly translated into an improvement in the microclimate, with a decrease in air humidity of approximately 4.5 percentage points and a decrease in CO_2_ levels of approximately 14% in the zeolite + EM group. According to Secondez and Barroga [70], spraying laying hen droppings with odor-erasing microbes (OEMs) resulted in a significant reduction in carbon dioxide levels in the poultry house. The litter additives used in our own research had no effect on the other greenhouse gases measured: methane and nitrous oxide. On the other hand, Pereira et al. [6] found a 34% reduction in N_2_O emissions after applying 2344 g/m^2^ of natural zeolite (clinoptilolite) to the litter, but showed no change in carbon dioxide and methane levels.

The environmental conditions in the broiler house have a significant impact on the productivity and welfare of broiler chickens. According to research by Hubert et al. [71], the litter itself can also influence the composition of the intestinal microbiome of chickens, their growth and health. In our own research, enriching the litter with zeolite and EMs improved the quality of the litter and air in the experimental broiler house, as well as increasing body weight, improving feed utilization, and improving the health of broiler chickens in this group. Other studies have also reported that the addition of zeolite to litter material has a positive effect on the growth and final body weight of birds [28,39,58,72]. In contrast, Basha et al. [73] and Schneider et al. [38] found no effect of adding 100 g/kg of zeolite to the litter on the body weight of broiler chickens throughout the rearing period. Similarly, Sujiwo and Ariyadi [74] and Abdulalwahhab and Al-Tememy [75] reported no increase in body weight in groups of birds reared on litter with zeolite inclusion. Pezzuolo et al. [76] tested the effect of probiotic bacteria in powder form as an additive to litter. The authors observed an increase in bird body weight at the end of the production cycle and a decrease in mortality rates. Studies on zeolite, on the other hand, report no effect of this litter additive on bird livability [28,74,75,77]. In our own studies, chicken mortality in the experimental group was almost half that of the CON group, which may have been influenced by the synergistic effect of the litter additives studied and the improvement in environmental conditions in this group. Eleroğlu and Yalçın [28] report that some zeolites have a positive effect on feed conversion efficiency, which may be related to the specific properties of particular zeolites or the particle size of the zeolites used. According to the authors, small zeolites added to litter can be consumed by birds, which can be linked to improved intestinal health by reducing the absorption of harmful chemicals, improving intestinal morphology, lowering pH, and reducing pathogenic microorganisms, resulting in better digestion of nutrients [13,78] and better feed efficiency. In studies by Eleroğlu and Yalçın [28], Ezenwosu et al. [39], Nikolakakis et al. [72] and Basha et al. [73], broiler chickens reared on litter with zeolite showed better feed efficiency than birds in the control group. Many studies also indicate that probiotics contribute to improved gut health by modulating the microflora, which has a positive effect on poultry production performance [79,80,81]. Wulf et al. [82] showed that litter can act as a carrier for probiotic bacteria, although their viability depends on the composition and quality of the litter. However, in their studies, they did not achieve improved production results (final body weight, feed intake, and feed conversion) by spraying the litter with a solution of distilled water and probiotic *Bacillus* spp. strains at a rate of 5 g/m^2^ before the birds were introduced. In contrast, our own research has shown that enriching the litter with 3 kg/10 kg of zeolite and simultaneously spraying the litter with a probiotic (EM) every week significantly improved feed utilization by broiler chickens, which translated into higher body weight.

Slaughter yield determines the commercial value of broiler chicken carcasses, and the most valuable parts of the carcass are the breast and leg muscles [83]. Naeem and Bourassa [84] report that the use of probiotics in poultry production has a beneficial effect on both yield and meat quality. In studies by Schneider et al. [38], Emam et al. [58], Basha et al. [73], and Banaszak et al. [85], no positive effect of zeolite, as an additive to bird litter, on slaughter yield and breast and leg muscles was found. In our own studies, the experimental factor zeolite + EM added to the litter had no effect on slaughter yield, but a higher percentage of breast muscle was obtained in the carcasses of experimental broilers, which is undoubtedly beneficial from an economic point of view.

One of the most important indicators of broiler meat quality is muscle acidity (pH), which determines physicochemical properties such as water-holding capacity (WHC), color, and meat texture [86,87,88]. Changes in water-holding capacity and drip loss can be correlated with changes in the pH level in meat [83]. As the pH decreases, the water-holding capacity, which determines the suitability of meat for processing, also decreases, as confirmed in our study for breast muscles. Improved water retention capacity of meat has a positive effect on further meat processing and contributes to the overall improvement of meat product quality [83,87]; hence, it can be concluded that broiler chickens from the zeolite + EM group had better breast muscle quality compared to CON birds.

The literature reports that lower pH values are associated with lighter meat color [86,89,90]. Our own research did not confirm this relationship, and no differences in L* values were found in the breast muscles of CON broiler chickens, even though they had a lower initial pH compared to birds from the zeolite + EM group. However, differences were noted for b* values. The breast muscles of broiler chickens from the CON group were less yellow (b*) compared to the breast muscles of birds from the zeolite + EM group. The color of skin and meat is a feature that consumers approach individually, also depending on the country and region, but they often base their product choice on it, thus assessing its quality and freshness [90,91,92].

In broiler chicken production, footpad dermatitis (FPD) is a very important problem. FPD negatively affects not only the welfare of the birds, but also the efficiency of rearing, broiler performance, and carcass quality [93]. High litter moisture is the main factor causing inflammatory changes on the soles of the feet and hock burns [94,95]. De Jong et al. [93], keeping broiler chickens on litter sprinkled with water to induce damage caused by very high substrate moisture, found that the average hock burn score was much worse in the induced group, and similar relationships were found for FPD. The improved litter quality resulting from the addition of zeolite + EM in our own studies had a positive effect on the quality of broiler chickens’ (FPD) and thus on their welfare, and the incidence of hock burns was also lower in this group of birds. In chickens kept on litter enriched with additives, the incidence of hock burns was significantly lower than the incidence of FPD. Kaukonen et al. [96] also showed that the incidence of hock burn lesions was much lower compared to the incidence of FPD. In our own studies, the average number of FPD points and inflammatory changes in the hock burns in the group of chickens kept on litter with zeolite + EM additive was lower compared to the control group by 29% and 32%, respectively. Wlaźlak et al. [97], using an additive of aluminosilicates, zeolite and halloysite (80:20), to wheat straw litter, found a beneficial effect of this additive on the quality of broiler chicken feet. Banaszak et al. [98], using zeolite as an additive to both feed and litter, demonstrated its beneficial effect on the condition of broiler chickens’ feet. However, Park et al. [40] obtained different results when keeping Ross 308 broiler chickens on litter with a 2% zeolite additive. These authors did not find a beneficial effect on FPD, despite obtaining lower litter moisture content. In contrast, Hussein and Areaaer [99] demonstrated the effect of adding zeolite to litter on improving the welfare of chickens, among other things, by reducing the incidence of FPD.

Additives to litter for broiler chickens improve its properties and at the same time reduce the load of microorganisms and parasites, improving the health of the birds’ feet, i.e., reducing the incidence of FPD [100]. According to Hidalgo et al. [66], one of the advantages of using EMs is the elimination of pathogenic organisms from the environment. Pedroso et al. [101] also showed that probiotics can alter the bacterial population of litter and reduce the incidence of certain pathogenic bacteria, which may indirectly reduce the incidence of secondary infections in broiler chickens. Probiotics not only stimulate poultry production performance, but can also have a beneficial effect on FPD, litter quality, and chicken manure emissions [62,102,103,104]. Park and Sun [53] demonstrated the effect of probiotic feed additives on reducing the incidence of FPD and lowering the average FPD score; similar results were obtained in our own studies. However, Flores et al. [105] found no effect of probiotics on the incidence of FPD.

It is known that changes in physiological parameters may be related to the health status of birds [13,106]. According to Zarnab et al. [107], elevated ammonia levels can lead to toxic damage to the kidneys and liver, which can lead to increased levels of AST, ALT, creatinine, and urea.

High creatinine levels may result from various kidney diseases, hepatorenal syndrome, reduced renal blood flow, or damage to various muscles [13]. According to Zarnab et al. [107], the weekly administration of 15 g/m^2^ of aluminum silicate nanoparticles to litter improved serum creatinine and urea concentrations in broiler chickens compared to the control group. In our own studies, the use of zeolite + EM in litter also reduced creatinine levels. However, Emam et al. [58] obtained different results: when using a 20% zeolite supplement in litter, they observed an increase in creatinine levels in Japanese quails. Elsherbeni et al. [13], using zeolite additives in the amounts of 0.5, 1, and 1.5 kg/m^2^ to sawdust litter, found a significant effect of zeolite on the increase in creatinine and ALAT levels. Elevated ALAT levels may indicate liver damage, e.g., by toxins [107]. Zhang et al. [108], studying the effect of high concentrations of ammonia in the air of broiler houses, demonstrated its effect on the increase in ALAT levels and confirmed that exposure to ammonia seriously impairs liver function. In our own studies, the additive used in the litter had a normalizing effect on liver function, as evidenced by reduced levels of ALAT and bilirubin, which is one of the indicators of liver function and red blood cell breakdown. Zarnab et al. [107] obtained similar results. These authors observed ALAT levels to decrease as a result of the use of aluminum silicate nanoparticles in litter for broiler chickens. Emam et al. [58] reported different results: using a 20% zeolite additive to the litter, they did not show its effect on ALAT levels. Mezzasalma et al. [109] demonstrated the effect of different litter materials on the levels of total cholesterol, HDL, bilirubin, and creatinine in the blood serum of broiler chickens, but found no changes in triglyceride, calcium, and ALAT levels on day 42 of the chickens’ lives. In our own studies, modifying the litter composition by adding zeolite + EM to improve litter quality and reduce ammonia emissions had a beneficial effect on liver indices, which led to a reduction in total cholesterol levels. Mohamed et al. [110] artificially induced high levels of ammonia in the air of broiler houses and demonstrated its effect on the increase in total cholesterol levels. Similarly, in our own studies, a decrease in NH_3_ concentration in the air was associated with a decrease in total cholesterol levels in the blood serum of broiler chickens.

At the same time, our own research observed a decrease in calcium and HDL concentrations and an increase in triglyceride concentrations in this group of birds. However, the calcium and triglyceride levels in both groups of birds studied were within the reference range given by Meluzzi et al. [111]. Golshahi et al. [81], using probiotic supplements in feed, found that they had an effect on reducing triglyceride, ALAT, and total cholesterol levels. Similarly, in our own studies, the zeolite + EM group showed reduced ALAT and total cholesterol levels. On the other hand, Kowalczyk et al. [112] applied the mineral–microbial product Deodoric (consisting, among others, of a mixture of six highly active bacterial strains) to the litter once a week and did not observe any effect on ALAT and calcium levels. However, these authors found an increase in creatinine and ASPAT concentrations in the group of chickens where a mineral–probiotic additive was applied to the litter. Monika et al. [113], using the probiotic *Lactobacillus acidophilus* (1 × 10^6^ cfu/g) in broiler feed, found no changes in total cholesterol, triglycerides, creatinine, and calcium levels.

Ammonia is released during protein metabolism, which is highly toxic to cells, but regulatory mechanisms in animals keep it below toxic levels. An excess or imbalance of amino acids in the diet causes the formation of ammonia, which is converted by birds into uric acid [114]. Emam et al. [58] used a 20% zeolite additive in litter for Japanese quails and demonstrated its effect on reducing blood ammonia levels by 37%. In our own studies, the addition of zeolite + EM to the litter resulted in a reduction in blood ammonia levels by approximately 24%, but no simultaneous increase in uric acid concentration was observed. In birds, ammonia is metabolized by conversion to uric acid; therefore, an increase in uric acid production should be an effective factor in reducing ammonia levels. Namroud et al. [114] demonstrated an increase in ammonia levels and a decrease in uric acid levels in the blood of broiler chickens fed with reduced-protein feed.

Overall, better litter quality and improved microclimatic conditions, thanks to the addition of zeolite + EM to the litter, improve bird comfort and reduce the risk of ammonia toxicity in the broiler house, leading to improved productivity and health. In addition, the proposed practice/method of broiler chicken production based on the addition of zeolite + EM to the litter allows for a reduction in the environmental impact of poultry farms in terms of limiting ammonia emissions.

Although the results of this study indicate a positive effect of zeolite + EM litter supplementation for broiler chickens, further research under large-scale production conditions is still needed. In addition, the research should be extended to include laying hens and egg quality, as well as an analysis of the quality of compost produced from such modified manure.

## 5. Conclusions

The results of this study indicate that the use of a zeolite additive in litter combined with weekly EM spraying improved litter quality (reduced pH and moisture), which had a positive effect on the microclimate parameters of broiler houses, primarily by reducing NH_3_ and CO_2_ emissions. The improved quality of the litter and air in the broiler house translated into improved production and health parameters of broiler chickens (including higher final body weight, better feed conversion, and lower mortality) without negatively affecting meat quality. Furthermore, the litter additive in the form of zeolite + EM used in the study had a positive effect on the welfare of the birds, as it improved the quality of the living environment of the broiler chickens (improvement in air parameters in the broiler house and litter quality), which was reflected in a lower incidence of FPD and hock burn inflammation.

This research supports the development of sustainable poultry production systems in Poland and is in line with EU climate goals. In summary, adding 3 kg/10 kg of zeolite to the litter while simultaneously spraying the litter with a 1:4 solution of EMs and water improves litter quality, reduces the emission of harmful gas admixtures produced on broiler farms, and has a positive effect on the productivity, health, and welfare of chickens.

## Figures and Tables

**Table 1:** Ingredient composition (%) and nutritive value of the diets.

Item	Diet			
	Starter ^1^ 1–9 Days	Grower 1 ^2^ 10–17 Days	Grower 2 ^3^ 18–28 Days	Finisher ^4^ 29–35 Days
Corn meal	57.04	60.09	63.405	65.61
Soybean meal	37	33.5	30	27.5
Rapeseed oil	2.3	3	3.2	3.8
Ground limestone	1.15	1	1	0.8
1-calcium phosphate	1.4	1.45	1.35	1.23
Sodium chloride	0.35	0.35	0.37	0.37
DL-Met (98%)	0.21	0.19	0.19	0.185
L-Threonine (98%)	-	-	0.035	0.03
Hydrochloride L-Lys (78%)	0.05	0.12	0.15	0.175
Vitamin–mineral premix starter SAL1 (0.5%) SA (0.3%)	0.5	-	-	-
Vitamin–mineral premix grower 1 ^2^ (0.3%)	-	0.3	-	-
Vitamin–mineral premix grower 2 ^3^ (0.3%)	-	-	0.3	-
Vitamin–mineral premix finisher ^4^ (0.3%)	-	-	-	0.3
Nutritive value per 1 kg (analyzed)				
Crude protein (%)	22.45	19.84	18.74	17.74
Metabolizable energy (MJ)	12.49	12.89	12.88	13.17
Lysine (g)	12.08	10.98	9.29	9.81
Methionine (g)	5.37	4.78	5.35	5.83
Calcium (g)	9.38	7.99	9.94	6.41
Phosphorus (g)	6.66	7.08	6.41	6.22

^1^ Supplements per kg starter diet: vit. A 12 500 IU; vit. D_3_ 5 000 IU; vit. E 125 mg; vit. B_1_ 3 mg; vit. B_2_ 8 mg; niacinamide (B_3_) 50 mg; vit. B_6_ 4 mg; vit. B_12_ 20 mcg; vit. K_3_ 3 mg; biotin 200 mcg; folic acid 2 mg; D-calcium pantothenate 16.33 mg; choline chloride 347.8 mg; Fe 25 mg; Mn 50 mg; Cu 10 mg; Zn 50 mg; I 1 mg; Se 0.35 mg; propyl gallate (E310) 0.15 mg; BHA 0.138 mg; BHT 1.91 mg; sepiolite 100 mg. ^2^ Supplements per kg grower 1 diet: vit. A 11 000 IU; vit. D_3_ 2 500 IU; vit. E 50 mg; vit. B_1_ 2 mg; vit B_2_ 7 mg; niacinamide (B_3_) 40; vit. B_6_ 4 mg; vit. B_12_ 21 mcg; vit. K_3_ 2.20 mg; biotin 201 mcg; folic acid 1 mg; choline chloride 300 mg; 25-hydroxy-cholecalciferol 0.0208 mg; Fe 45 mg; Mn 70 mg; Cu 20 mg; Zn 95 mg; Se 0.35 mg; propyl gallate (E310) 0.09 mg; BHA (E320) 0.083 mg; BHT (E321) 1.15 mg; D-calcium pantothenate 13.61 mg. ^3^ Supplements per kg grower 2 diet: vit. A 10 000 IU; vit. D_3_ 3 000 IU; vit. E 30 mg; vit. B_1_ 2.51 mg; vit B_2_ 5.01 mg; niacinamide (B_3_) 35.01 mg; vit. B_6_ 3.51 mg; vit. B_12_ 21 mcg; vit. K_3_ 2.51 mg; biotin 201 mcg; folic acid 1 mg; choline chloride 300 mg; Fe 20 mg; Mn 40 mg; Zn 45 mg; propyl gallate (E310) 0.09 mg; BHA (E320) 0.083; mg; BHT (E321) 1.15 mg; D-calcium pantothenate 13.07 mg; sepiolite (E562) 150 mg. ^4^ Supplements per kg finisher diet: vit. A 10 000 IU; vit. D_3_ 2 000 IU; vit. E 30 mg; vit. B_1_ 2 mg; vit B_2_ 6 mg; niacinamide (B_3_) 40 mg; vit. B_6_ 3 mg; vit. B_12_ 21 mcg; vit. K_3_ 2 mg; biotin 150 mcg; choline chloride 300 mg; folic acid 1 mg; Fe 20 mg; Mn 35 mg; Cu; 10 mg; Zn 25 mg; I 0.50 mg; Se 0.20 mg; propyl gallate (E310) 0.09 mg; BHA (E320) 0.083 mg; BHT (E321) 1.15 mg; D-calcium pantothenate 10.89 mg; sepiolite (E562) 60 mg.

**Table 2:** pH * and moisture * of the litter in broiler house during the rearing of broiler chickens (*n* = 6 per group (replicate—pen)).

Item	Week of Rearing	Group				
		CON	SD	Zeolite + EM	SD	*p*-Value
pH	1	6.56	0.26	6.50	0.52	0.934
	2	7.69	0.28	7.36	0.25	0.052
	3	8.05 ^A^	0.13	7.50 ^B^	0.92	0.008
	4	9.13 ^A^	0.10	8.81 ^B^	0.14	0.001
	5	9.24 ^a^	0.03	8.96 ^b^	0.06	0.015
Average pH of the litter	1–5	8.13 ^a^	0.91	7.83 ^b^	0.86	0.013
Moisture (%)	1	15.17	3.90	10.11	1.20	0.784
	2	31.82	20.75	25.20	7.88	0.837
	3	41.92 ^a^	24.67	29.18 ^b^	20.48	0.036
	4	50.88 ^a^	25.82	34.00 ^b^	21.64	0.026
	5	70.96 ^A^	19.99	55.90 ^B^	22.41	0.007
Average moisture of the litter (%)	1–5	42.15 ^a^	33.98	30.88 ^b^	21.55	0.036

^a,b^—values in rows with different letters differ significantly (*p* < 0.05); ^A,B^—values in rows with different letters differ highly significantly (*p* < 0.01); CON—control group; SD—Standard Deviation; EM—effective microorganism (probiotic); * litter sampled from 6 pens (subgroups) in every week from 5 different locations.

**Table 3:** Average air quality * in broiler house during the rearing of broiler chickens (*n* = 6 per group (replicate—pen)).

Item	Group				
	CON	SD	Zeolite + EM	SD	*p*-Value
Temperature °C	23.87	2.21	24.01	2.06	0.760
Humidity (%)	69.80 ^A^	8.99	65.21 ^B^	4.94	0.004
NH_3_ (ppm)	4.63 ^a^	5.21	2.13 ^b^	3.22	0.039
CO_2_ (ppm)	1415.73 ^a^	629.47	1216.63 ^b^	489.88	0.031
CH_4_ (ppm)	1.78	0.21	1.62	0.04	0.092
N_2_O (ppm)	0.109	0.01	0.113	0.01	0.161
CO (ppm)	0.391	0.10	0.364	0.13	0.280

^a,b^—values in rows with different letters differ significantly (*p* < 0.05); ^A,B^—values in rows with different letters differ highly significantly (*p* < 0.01); CON—control group; SD—Standard Deviation; EM—effective microorganism (probiotic); * measurements were taken twice a week in 6 pens (subgroups) at 5 locations.

**Table 4:** Body weight (*n* = 240 per group), feed conversion (*n* = 6 per group (replicate—pen)), and mortality of broiler chickens between days 1 and 35 of rearing.

Item	Days of Rearing	Group				
		CON	SD	Zeolite + EM	SD	*p*-Value
Body weight (g)	1	47.07	3.98	46.47	3.83	0.263
	9	179.88 ^a^	33.10	190.79 ^b^	35.96	0.047
	16	517.65 ^A^	92.80	554.44 ^B^	88.75	0.002
	28	1439.94	280,10	1477.13	252.74	0.214
	35	2326.27 ^a^	363.35	2388.14 ^b^	365.06	0.015
Feed conversion (g/kg b.w.)	1–35	1960.00 ^a^	82.55	1860.00 ^b^	71.43	0.013
Mortality (%)	1–35	6.55 ^a^	3.51	3.57 ^b^	4.52	0.023

^a,b^—values in rows with different letters differ significantly (*p* < 0.05); ^A,B^—values in rows with different letters differ highly significantly (*p* < 0.01); CON—control group; SD—Standard Deviation; EM—effective microorganism (probiotic).

**Table 5:** Slaughter analysis results (%) (*n* = 20 birds per group).

Item	Group				
	CON	SD	Zeolite + EM	SD	*p*-Value
Dressing percentage with giblets	75.59	1.97	74.88	2.48	0.348
Dressing percentage without giblets	72.49	2.05	71.73	2.49	0.326
Breast muscles	24.81 ^a^	2.58	26.69 ^b^	2.11	0.023
Leg muscles	20.60	1.41	20.42	1.75	0.736
Leg bones	4.93	0.28	4.74	0.50	0.167
Abdominal fat	2.61	0.47	2.34	0.56	0.139
Giblets	4.10	0.41	4.21	0.41	0.461
Liver	3.69	5.24	2.50	0.26	0.344
Gizzard	0.91	0.28	1.03	0.18	0.139
Heart	0.69	0.13	0.68	0.09	0.918

^a,b^—values in rows with different letters differ significantly (*p* < 0.05); CON—control group; SD—Standard Deviation; EM—effective microorganism (probiotic).

**Table 6:** pH of breast and leg muscles (*n* = 20 breast and 20 leg muscles per group).

Item		Group				
		CON	SD	Zeolite + EM	SD	*p*-Value
Breast muscles	pH_15_	6.36 ^A^	0.19	6.56 ^B^	0.13	0.010
	pH_24_	5.81	0.10	5.79	0.12	0.300
Leg muscles	pH_15_	6.61	0.19	6.48	0.20	0.743
	pH_24_	6.18	0.18	6.21	0.13	0.067

^A,B^—values in rows with different letters differ highly significantly (*p* < 0.01); CON—control group; SD—Standard Deviation; EM—effective microorganism (probiotic); pH_15_—pH of breast and leg muscles measured fifteen minutes post-mortem; pH_24_—pH of breast and leg muscles measured 24 h post-mortem.

**Table 7:** Drip loss and water-holding capacity of meat (*n* = 20 samples of breast muscles and 20 of leg muscles per group).

Item		Group				
		CON	SD	Zeolite + EM	SD	*p*-Value
Breast muscles						
Drip loss (%)	24 h of cooling	2.00	0.86	1.93	0.64	0.764
	48 h of cooling	3.59	1.46	3.42	1.11	0.691
	31 days of freezing	9.13	3.38	9.49	3.08	0.750
WHC (%)		28.88 ^A^	2.09	24.38 ^B^	3.76	0.004
Leg muscles						
Drip loss (%)	24 h of cooling	0.71	0.17	0.83	0.44	0.287
	48 h of cooling	1.22	0.36	1.10	0.53	0.320
	31 days of freezing	5.94	2.29	5.08	2.19	0.265
WHC (%)		24.24	4.06	24.10	4.86	0.945

^A,B^—values in rows with different letters differ highly significantly (*p* < 0.01); CON—control group; SD—Standard Deviation; EM—effective microorganism (probiotic); WHC—water-holding capacity.

**Table 8:** Color of breast and leg muscles (*n* = 20 breast and 20 leg muscles per group).

Item		Group				
		CON	SD	Zeolite + EM	SD	*p*-Value
Breast muscles	L*	61.28	2.14	61.18	2.08	0.244
	a*	10.92	1.32	10.86	1.22	0.299
	b*	11.12 ^a^	1.54	11.81 ^b^	1.19	0.013
Leg muscles	L*	57.61	4.38	57.74	4.45	0.271
	a*	16.01	1.62	15.77	1.76	0.409
	b*	10.27	1.44	10.81	1.34	0.508

^a,b^—values in rows with different letters differ significantly (*p* < 0.05); CON—control group; SD—Standard Deviation; EM—effective microorganism (probiotic); the color on the CIE L*a*b* scale: L* represents lightness, a* redness, and b* yellowness.

**Table 9:** Condition of feet of broiler chickens 0–2 points (*n* = 60 birds per group).

Item	Group				
	CON	SD	Zeolite + EM	SD	*p*-Value
^1^ FPD					
0 (%)	0.00	0.01	5.55	13.61	0.341
1 (%)	63.83	12.54	61.11	20.18	0.780
2 (%)	36.17	14.89	33.34	12.52	0.729
Average points	1.36 ^a^	0.61	0.96 ^b^	0.28	0.014
^1^ Changes in the hock burns					
0 (%)	8.35 ^A^	9.15	47.78 ^B^	22.18	0.004
1 (%)	58.3	20.17	52.22	9.15	0.670
2 (%)	33.35 ^A^	18.24	0.00 ^B^	0.008	<0.001
Average points	1.06 ^a^	0.79	0.72 ^b^	0.24	0.032

^A,B^—values in rows with different letters differ highly significantly (*p* < 0.01); ^a,b^—values in rows with different letters differ significantly (*p* < 0.05); CON—control group; SD—Standard Deviation; EM—effective microorganism (probiotic); FPD—footpad dermatitis. ^1^ 0 = No lesions: no or very small superficial lesions, slight discoloration on a limited area, mild hyperkeratosis. 1 = Mild lesion: discoloration of the foot pad, superficial lesions, dark papillae. 2 = Severe lesion: ulcers or scabs, signs of hemorrhages or swollen foot pads.

**Table 10:** Blood parameters of broiler chickens (*n* = 10 birds per group).

Item	Group				
	CON	SD	Zeolite + EM	SD	*p*-Value
Ammonia (umol/L)	72.47 ^A^	12.52	55.28 ^B^	8.61	0.002
ALAT (U/L)	2.22 ^a^	1.00	1.50 ^b^	0.79	0.022
ASPAT (U/L)	207.50	43.38	210.44	55.43	0.860
ALP (U/L)	2123.39	660.22	2359.67	589.67	0.265
LDH (U/L)	1248.50	497.55	890.78	825.16	0.125
Bilirubin (mg/dL)	0.30 ^a^	0.09	0.23 ^b^	0.11	0.026
Glucose (mg/dL)	275.72	30.34	266.33	41.56	0.444
Ca (mg/dL)	13.50 ^A^	2.63	11.38 ^B^	1.03	0.003
P (mmol/L)	2.33	0.29	2.32	0.31	0.951
Fe (ug/dL)	54.13	26.25	62.81	15.89	0.238
Creatinine (mg/dL)	0.35 ^A^	0.04	0.30 ^B^	0.05	0.002
Urea (mg/dL)	17.56	1.13	17.89	0.99	0.352
Uric acid (mg/dL)	4.24	1.23	4.03	1.10	0.589
Total cholesterol (mg/dL)	152.06 ^a^	15.10	142.28 ^b^	13.62	0.049
HDL (mg/dL)	79.83 ^a^	8.87	73.61 ^b^	6.78	0.024
LDL (mg/dL)	56.44	12.43	56.27	22.66	0.340
Triglycerides (mg/dL)	45.80 ^A^	9.25	61.67 ^B^	10.13	0.005
NA (mmol/L)	153.34	5.25	154.51	4.17	0.465
K (mmol/L)	5.41	0.40	5.19	0.50	0.153

^a,b^—values in rows with different letters differ significantly (*p* < 0.05); ^A,B^—values in rows with different letters differ highly significantly (*p* < 0.01); CON—control group; SD—Standard Deviation; EM—effective microorganism (probiotic).

## Data Availability

The data presented in this study are available on request from the authors.

## Abbreviations

The following abbreviations are used in this manuscript:

CH_4_	Methane
CO_2_	Carbon dioxide
EM	Effective microorganism
FPD	Footpad dermatitis
GHG	Greenhouse gas
NH_3_	Ammonia
N_2_O	Nitrous oxide
WHC	Water-holding capacity
